# Using Single-Cell RNA-Seq Data to Trace Tissue Cells Responsive to Thyroid Hormones

**DOI:** 10.3389/fendo.2021.609308

**Published:** 2021-02-24

**Authors:** Liang Hu, Chao Wu

**Affiliations:** ^1^ Department of Thyroid Surgery, The First Affiliated Hospital, Zhejiang University School of Medicine, Hangzhou, China; ^2^ State Key Laboratory for Diagnosis and Treatment of Infectious Diseases, National Clinical Research Center for Infectious Diseases, Collaborative Innovation Center for Diagnosis and Treatment of Infectious Diseases, The First Affiliated Hospital, Zhejiang University School of Medicine, Hangzhou, China

**Keywords:** thyroid hormone, Thra/Thrb, single-cell RNA-Seq, bioinformatics analysis, cell-type-specific expression

## Abstract

Thyroid hormones mediate a remarkable range of functions in many tissues and organ systems through the thyroid hormone receptors—THRA and THRB. Tissues and organs are composed of heterogeneous cells of different cell types. These different cell types have varying receptor expression abilities, which lead to variable responses in thyroid hormone regulation. The tissue-specific Thra and Thrb gene expression patterns help us understand the action of thyroid hormones at the tissue level. However, the situation becomes complicated if we wish to focus on tissues more closely to trace the responsive cells, which is a vital step in the process of understanding the molecular mechanism of diseases related to thyroid hormone regulation. Single-cell RNA sequencing technology is a powerful tool used to profile gene expression programs in individual cells. The Tabula Muris Consortium generates a single-cell transcriptomic atlas across the life span of *Mus musculus* that includes data from 23 tissues and organs. It provides an unprecedented opportunity to understand thyroid hormone regulation at the cell type resolution. We demonstrated the approaches that allow application of the single-cell RNA-Seq data generated by the Tabula Muris Consortium to trace responsive cells in tissues. First, employing the single-cell RNA-Seq data, we calculated the ability of different cell types to express Thra and Thrb, which direct us to the cell types sensitive to thyroid hormone regulation in tissues and organs. Next, using a cell clustering algorithm, we explored the subtypes with low Thra or Thrb expression within the different cell types and identified the potentially responsive cell subtypes. Finally, in the liver tissue treated with thyroid hormones, using the single-cell RNA-Seq data, we successfully traced the responsive cell types. We acknowledge that the computational predictions reported here need to be further validated using wet-lab experiments. However, we believe our results provide powerful information and will be beneficial for wet lab researchers.

## Introduction

Thyroid hormones mediate a remarkable range of functions in many tissues and organ systems (1). Much of the action of thyroid hormones is mediated by thyroid hormone receptors (TRs), which act as ligand-regulated transcription factors and occupy a key position in the chain of events that produce cellular responses (2). There are two main classes of thyroid hormone receptors: alpha, which is encoded by THRA and beta, which is encoded by THRB (3). The ability to express either of the receptor genes in a cell provides a means of conferring a specific biological response towards thyroid hormone regulation.

Over the last three decades, numerous studies related to the expression of thyroid hormone receptor genes have been published. Forrest et al. compared the expression of TR alpha and TR beta mRNAs during chicken development until three weeks post-hatching in 14 different tissues (4). Bookout et al. investigated the expression of Thra and Thrb, which are the genes analogous to THRA and THRB in mice in 39 different tissues from two strains of mice, 12931/SvJ and C57/Bl6 and found widespread tissue expression (5). Chassande et al. observed that Thra is expressed at the very beginning of mouse development (6). Bradley et al. examined TR gene expression in 12 stages of the developing rat’s nervous system and found high expression of Thra in neural tissues (7). The expression of Thra and Thrb has been thoroughly investigated; Flamant and Gauthier provided a comprehensive review of this topic (2). In humans, mutations in the thyroid hormone receptor gene are associated with thyroid hormone resistance (8), which has resulted in substantial work in laboratories across the world to investigate their expression in multiple tissues. Kublaoui and Levine summarized the expression patterns of THRA and THRB in human tissues (9). In summary, TR genes are expressed in most tissues in mice and humans. However, THRA/Thra is predominantly expressed in the heart and brain, whereas THRB/Thrb is largely expressed in the brain, fat, kidney, and liver.

The research discussed in the previous paragraph has provided us with an understanding of the expression pattern of TR genes in tissues. However, tissues are comprised of multiple cell types. The situation becomes complicated when we wish to focus on tissue more closely to trace the responsive cells, which is a vital step in the process of understanding the molecular mechanism of diseases related to thyroid hormone regulation. *In vitro* cell culture has been applied to study TR gene expression and function in chondrocytes, hepatocytes, neurons, and other cell types (2). However, concerns around the ability of an *in vitro* cell culture to mimic *in vivo* cells exist. Single-cell RNA sequencing (RNA-Seq) technology is a powerful tool used to profile the gene expression programs in an individual cell (10). It provides an unprecedented opportunity to capture the transcriptome of any cell *in vivo*. The massive sequencing of single cells from multiple tissues of human and model animals, such as the Tabula Muris Consortium and the mouse cell atlas (MCA) project, paints the portraits of cells in the tissues and significantly improves our understanding of the transcriptome characterization of each cell type, especially the previously poorly characterized ones (11–14). By employing these data resources, we are able to comprehensively detect the tissues and organ cells that express Thra or Thrb as well as understand the expression pattern of TR genes in different cell types.

Here, using the single-cell RNA-Seq data from the Tabula Muris Consortium, we calculated Thra and Thrb expression in 101 cell types. We found that brain cells, including oligodendrocytes, medium spiny neurons, neuronal stem cells, neurons, and brain pericytes, exhibited the most significant Thra expression while medium spiny neurons, hepatocytes, ventricular myocytes exhibited the most significant Thrb expression. Next, the cell types that exhibited low Thra or Thrb expression were divided into potential subtypes. In myeloid cells, we found that 20% of cells in the Stab1 marker subtype and less than 2% of cells in the Cd300e marker subtype expressed Thra, which implies they have different capacities to respond to thyroid hormone regulation triggered by Thra. Finally, we integrated the bulk RNA-Seq data from mouse liver cells with the short- or long-term triiodothyronine (T3) treatment and the single-cell RNA-Seq data of the different cell types comprising the liver. We found that hepatocytes primarily responded to the short- and long-term T3 treatment and that immune cells also responded during the long-term T3 treatment. The above results were obtained following *in silico* analyses, and further validation using wet lab experiments is required. However, it provides approaches to trace tissue cells responsive to thyroid hormones using single-cell RNA-Seq data from Tabula Muris Consortium.

## Materials and Methods

### Data

The single-cell RNA-seq data generated by the Tabula Muris Consortium from the GEO database were downloaded (15). For cells from the SMART-Seq2 platform, we removed the cells with fewer than 5,000 counts and 500 detected genes. For cells from the 10× Genomics platform, we removed the cells with fewer than 2,500 unique molecular identifiers (UMIs) and 500 detected genes. The cell annotation file for the cells was also downloaded. The bulk tissue RNA-Seq data for 17 tissues were obtained from the GEO database. This includes the raw profiles of 54,352 genes for 947 samples. The tissue annotation file for the samples was also obtained. The liver tissue RNA-Seq data for long-term thyroid hormone treated mouse samples and controls were downloaded from the GEO database. This includes the raw profiles of 13,319 genes for eight mice samples. Information on the downloaded files is provided in **Table S9**.

### Gene Expression Processing

For the liver tissue RNA-Seq data, the RPM (reads per million mapped reads) of each gene as its expression value were calculated. Using the single-cell RNA-Seq data of the ten cell types, including: natural killer (NK) cells; B cells; T cells; neutrophils; macrophages; Kupffer cells; hepatocytes; endothelial cells of the hepatic sinusoid; fibroblasts; and, pancreatic stellate cells, we calculated the percentage gene expression profiles as follows: First, for each gene and cell type, the number of cells expressing each gene in each cell type were counted and the percentage of cells in each cell type that express each gene was calculated; second, the calculated percentages were taken as the expression level of each gene in each cell type; finally, the expression levels for all 22,966 genes in each of the ten cell types were obtained *via* this method.

### Gene Co-Expression Analysis

We employed the hypergeometric test to test the co-expression of TRs genes and transporters (or deiodinases) in each cell type. Supposed *n* is the number of total sequenced cells, m is the number of cells expressing A gene, k is the number of cells expressing B gene, if m > 9 and k > 9, we calculated the possibility (p) of finding x or more than x cells expressing both A and B genes randomly using R function phyper as follows,

p=1-phyper((x-1),m,(n-m),k).

### Using the Seurat Package to Group Cells Into Cell Clusters and Identify Marker Genes for Each Cluster

#### Single-Cell RNA-Seq Data Processing

The single-cell RNA-Seq data from the three-month time point derived from the SMART-Seq2 RNA-Seq libraries in the Tabula Muris Consortium were extracted. The data comprise single-cell transcriptomic data from 44,518 annotated cells. Next, the Seurat (version 3.1.5) package was employed to conduct the following analyses. The “NormalizeData” function was used to remove the differences in sequencing depth across the cells. The “normalized.method” parameter was set as “LogNormalize” and the “scale.factor” parameter was 10,000.

#### Dimension Reduction

Dimension reduction was performed at three stages of the analysis: the selection of variable genes, PCA, and UMAP. The “FindVariableFeatures” function was applied using the “selection.method” parameter set as “mean.var.plot” to select the highly variable genes that cover most of the biological information within the transcriptome. Then, the variable genes for PCA were implemented using the “RunPCA” function. Next, the principal components 1–15 were selected as inputs to perform the “RunUMAP” function and obtain bidimensional coordinates for each cell.

#### Unsupervised Clustering and Annotation

The “FindClusters” function was performed to cluster the cells. The “resolution” parameter was set at 0.5 and the default settings were used for the other parameters. Next, the “RunUMAP” and “DimPlot” functions were employed using the default parameters to obtain the cell clusters plots.

#### Identification of Marker Genes

The “FindAllMarkers” function was used to identify the marker genes based on the normalized data. The Wilcoxon rank-sum test was employed to identify the genes with at least a two-fold fold change and with a detectable percentage (at least 20%) in either cluster. A p-value adjustment was performed using the Bonferroni correction based on the total number of genes in the dataset. Marker-genes with adjusted p-values >0.05 were filtered out.

### Gene Differential Expression Analysis

The DEGs for the samples treated with T3 and the controls using DESeq2 were determined. The downloaded raw profiles were inputted, and the default parameters were used to obtain DEGs with false discovery rate values <0.05.

### Clustering Analysis of the DEGs

The percentage expression profiles in each of the ten cell types were obtained for the inputted genes. The R package “factoextra” was employed to conduct the clustering analysis (16). The “Euclidean” method was used to measure the distance between the observations, the “ward.D2” method was selected for agglomeration of the observations, and the “fviz_dend” function was employed for dendrogram visualization. The clustering tree was cut into groups. Finally, the genes were sorted according to the clustering results and a heatmap of the gene expression levels was produced.

### GO Term Enrichment Analysis

A GO term enrichment analysis was conducted on the selected genes. The analysis tool used was DAVID 6.8 (17). The GO terms from the “GOTERM_BP_DIRECT” ontology, which have a Bonferroni corrected p-value <0.05, were used.

## Results

### Thra and Thrb Expression Using Single-Cell RNA-Seq Data Generated by the Tabula Muris Consortium

The Tabula Muris Consortium performed single-cell RNA sequencing using a SMART-Seq2 platform and a 10× Genomics platform on more than 350,000 cells from male and female C57BL/6JN mice belonging to six age groups, ranging from one month (the equivalent of human early childhood) to 30 months (the equivalent of a human centenarian) (14). We selected cells that have detected more than 500 genes and provided cell-type annotation information. Thus, from sequencing using the SMART-Seq2 platform, we obtained 44,518 cells from three-month-old mice, 34,027 cells from 18-month-old mice, and 31,551 cells from 24-month-old mice. In addition, using the 10× Genomics platform we obtained 61,808 cells from one-month-old mice, 45,602 cells from three-month-old mice, 44,645 cells from 18-month-old mice, 35,828 cells from 21-month-old mice, 37,660 cells from 24-month-old mice, and 55,674 cells from 30-month-old mice. For each platform used, the cells from each group were classified by cell type, and the cell types present in numbers of 20 more cells were selected. The number of cells expressing Thra or Thrb was counted, and the percentage of cells expressing Thra or Thrb in each cell type was calculated, which represented the level of Thra or Thrb expression in each cell type.

We found 78 cell types present in numbers of more than 20 cells in all three-,18-, and 24-month-old mice sequenced using the SMART-Seq2 platform. We compared Thra and Thrb expression in the cell types (**Figure S1A**). The top five cell types ranked by Thra expression in 3-month-old mice are oligodendrocytes, neurons, brain pericytes, bladder cells, and mesenchymal stem cells of adipose tissue; those in 18-month-old mice are oligodendrocytes, pancreatic ductal cells, epidermal cells, mesenchymal stem cells of adipose tissue, and valve cells; and those in 24-month-old mice are bladder cells, neurons, mesenchymal stem cells of adipose tissue, brain pericytes, and oligodendrocytes. The results suggest that oligodendrocytes, neurons, and brain pericytes from the brain have significant Thra expression in the three ages of mice studied. Although the top cell types in 18-month-old mice are different from those in the three- and 24-month-old mice, the correlation analysis shows that the Thra expression pattern in the 78 cell types is similar between the different ages of mice (**Figure S1B**). The top five cell types ranked by Thrb expression in three-month-old mice are hepatocytes, intestinal crypt stem cells, bladder urothelial cells, secretory cells, and pancreatic D cells; those in 18-month-old mice are secretory cells, intestinal crypt stem cells, pancreatic A cells, bladder urothelial cells, and pancreatic D cells; and those in 24-month-old mice are hepatocytes, secretory cells, intestinal crypt stem cells, Kupffer cells, and epidermal cells. The results suggest that hepatocytes, intestinal crypt stem cells, secretory cells, pancreatic D cells have significant Thrb expression in the three ages of mice. The correlation analysis also shows that the Thrb expression pattern is similar between the different ages of mice (**Figure S1B**).

We observed 30 cell types in numbers of 20 or more cells in all one-, three-, 18-, 21, 24-, and 30-month-old mice sequenced using 10× Genomics platform. Most of the cell types are immune cells and have relatively low Thra and Thrb expression (**Figure S2A**). In the six age groups of mice, fibroblasts of the cardiac tissue and endocardial cells have the highest Thra expression, while hepatocytes have the highest Thrb expression. The correlation analysis shows that the patterns of Thra and Thrb expression in the 30 cell types are similar between the different ages of mice (**Figure S2B**).

The SMART-Seq2 platform sequences full-length mRNAs, while the 10× Genomics platform sequences mRNA with UTR fragments, which suggests the former platform provides higher sensitivity and coverage for gene detection. The work released by the Tabula Muris Consortium showed that, for cells from the same tissue, more genes were detected using the SMART-Seq2 platform than that using the 10× Genomics platform [extended data **Figure 5A** in (18)]. The Tabula Muris Consortium used both the SMART-Seq2 platform and the 10× Genomics platform to sequence cells from three-, 18-, and 24-month-old mice. We compared Thra and Thrb expression in the data generated by the two platforms. We found 56 cell types in numbers of 20 or more cells in three-month-old mice using both platforms. Similarly, we found 61 cell types and 29 cell types in 18- and 24-month-old mice. The correlation coefficients of Thra (or Thrb) expression over the cell types using the different platforms are high for the three age group mice (**Figure S3**). The correlation analysis shows that Thra (or Thrb) expression patterns are consistent between the different platforms (**Figure S3**). However, we found that the SMART-Seq2 platform tended to report the higher Thra and Thrb expression than the 10× Genomics platform for most cell types analyzed (**Figure S3**), demonstrating the advantage of the SMART-Seq2 platform on sensitivity for gene detection.

Overall, the SMART-Seq2 and 10× Genomics platforms reported similar Thra and Thrb expression patterns over different cell types. The expression patterns of Thra and Thrb over different cell types are not largely affected by ages. However, the SMART-Seq2 platform outperforms the 10× Genomics platform on detecting Thra and Thrb expression in cells with higher sensitivity.

### Thra and Thrb Expression in 101 Cell Types From Three-Month-Old Mice

As an example, we took the single-cell RNA-Seq data generated by the SMART-Seq2 platform from three-month-old mice to explicate Thra and Thrb expression in the cell types. We analyzed the data from three-month-old mice because three-month mice are the preferred model for studying thyroid hormone regulation in tissues over 18- and 24-month-old mice. We detected 101 cell types present in numbers of more than 20 cells using the SMART-Seq2 platform in three-month-old mice (**Table S1**). Using the 10× Genomics platform, 85 cell types present in numbers of more than 20 cells were detected in three-month-old mice. Of the cells detected using the 10× Genomics platform, 29 of the 85 cell types were unique, including many cell types from the kidney. The Thra and Thrb expression in the 29 cell types is shown in **Figure S4**. Integration of the results of Thra and Thrb expression from the two platforms was not performed because they measure gene expression in significantly different ways. Next, we focused on Thra and Thrb expression in the 101 cell types in three-month-old mice using the SMART-Seq2 platform.

**Figure 1:**
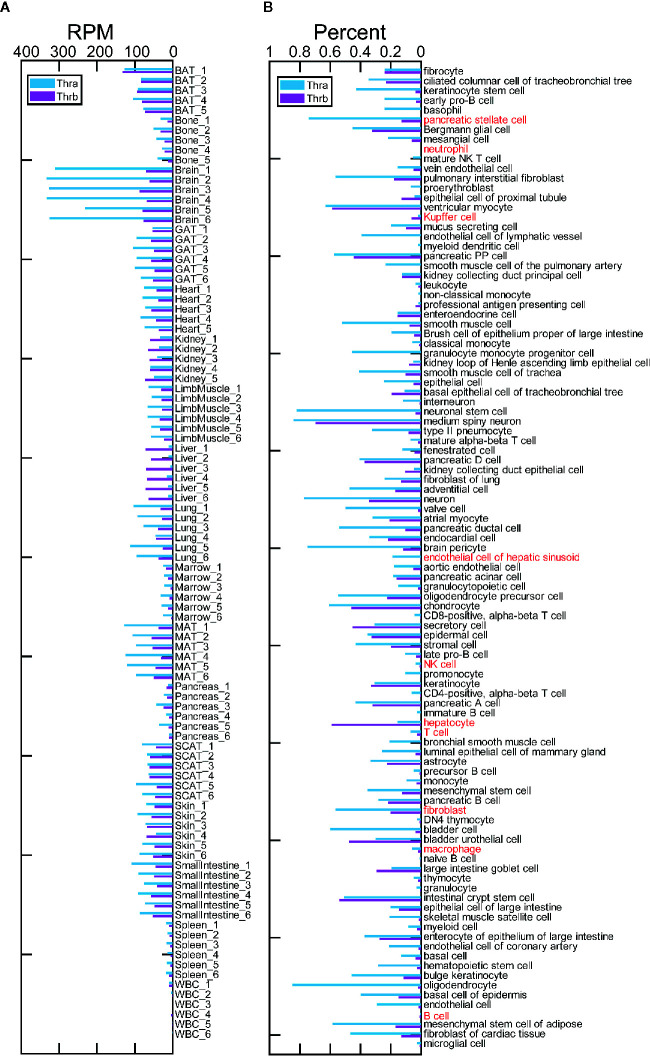
Tissue and cell type expression of Thra and Thrb in three-month-old mice. **(A)** The expression of Thra and Thrb in the samples from 17 tissues and organs. RPM, reads per million mapped reads; BAT, brown adipose tissue; GAT, gonadal adipose tissue; MAT, mesenteric adipose tissue; SCAT, subcutaneous adipose tissue; WBC, white blood cells. **(B)** The expression percentage of Thra and Thrb in 101 cell types. The cell types used to simulate the liver cell microenvironment are highlighted in red.

The Tabula Muris Consortium provides tissue bulk RNA-Seq profiles from 17 organs from two female and four male, C57BL/6JN, three-month-old mice. We calculated the RPM (reads per million mapped reads) of Thra and Thrb for the samples from tissue bulk RNA-Seq data (**Figure 1A**). We found that Thra exhibits a clear, high expression in the brain and that Thrb is also highly expressed in the brain, fat, kidney, and liver. In most of the tissues studied, Thra exhibits higher expression than Thrb. However, in liver tissue, Thrb is more highly expressed than Thra. These results are consistent with previous studies of tissue-specific expression patterns for Thrb and Thra. We detected Thra and Thrb expression in the 101 cell types (**Figure 1B**). We ranked the cell types by their percentage of Thra expression and found that the top five cell types are oligodendrocytes (0.85), medium spiny neurons (0.84), neuronal stem cells (0.82), neurons (0.77), and brain pericytes (0.74), which may explain why Thra is expressed to a substantially higher level in the brain. We ranked the cell types by their percentage of Thrb expression and found the top cell types are medium spiny neurons (0.70), hepatocytes (0.59), and ventricular myocytes (0.59).

We simulated the cell microenvironment of the liver tissue using ten cell types, including: natural killer (NK) cells, B cells, T cells, neutrophils, macrophages, Kupffer cells, hepatocytes, endothelial cells of the hepatic sinusoid, fibroblasts, and pancreatic stellate cells. Hepatic stellate cells are significant players in liver function (19). However, the hepatic stellate cells are out of the 101 cell types. We found ample evidence to demonstrate that pancreatic stellate cells and hepatic stellate cells display similar functions (20). With that, we added pancreatic stellate cells in place of the hepatic stellate cells. We took the gene expression profiles of the ten cell types to simulate the expression profiles of liver cells (see *Gene Expression Processing* in the *Materials and Methods*). We found that immune cells have extremely low Thra and Thrb expression (**Figure 1B**). Fibroblasts show the most significant Thra expression. We found that in nine of the cell types, neither the Thra nor Thrb gene is expressed, or Thra is expressed at a higher percentage than Thrb (**Figure 1B**). However, in the hepatocytes, Thrb is expressed at a significantly higher percentage than Thra. In the liver tissue, Thrb is expressed at a higher level than Thra (**Figure 1A**). As hepatocytes make up 55–65% of the liver mass (21), they dominate the response to thyroid hormone regulation through Thrb in liver tissue.

Previous studies have demonstrated that cellular responses to thyroid hormones are not only dependent on the presence of TRs but also on the appearance of thyroid hormone transporters Slc16a2 (also known as Mct8), Slc16a10 (also known as Mct10), and Slco1c1 (also known as Oatp14) and hormone availability, which is determined either by the activation of thyroxine (T4) into triiodothyronine (T3) by deiodinases Dio2 or by the inactivation of T4 into reverse T3 by deiodinases Dio3 (22, 23). We also calculated the percentage of cells expressing these genes in each of the 101 cell types and took the percentage as the value of their expression (**Figure S5**). We found that Slco1c1 expression is low in all cell types, suggesting it is not detected. The single-cell RNA-Seq data give us a chance to study the co-expression between genes in individual cells. We analyzed co-expression between TR genes and transporters (or deiodinases) in each of the 101 cell types using the hypergeometric test (see *Gene Co-Expression Analysis* in the *Materials and Methods*).

Dio2 and Dio3 expression was detected in only a few cells in most cell types. We successfully analyzed the co-expression between Thra and Dio2 in 19 cell types and between Thrb and Dio2 in 16 cell types (**Table S2**). With p <0.05, we found Thra and Thrb both tended to be co-expressed with Dio2 in astrocytes, which suggests T4 tends to be transformed into T3 in Thra- and Thrb-expressing astrocytes. We successfully analyzed the co-expression between Thra and Dio3 in three cell types and between Thrb and Dio3 in three cell types (**Table S2**). With p <0.05, Thra tended to be co-expressed with Dio3 in mesenchymal stem cells of adipose tissues. Although mesenchymal stem cells of adipose tissues have a high Thra expression (0.59), the result suggests the Thra-expressing cells exhibit the capability of inactivation of T4 into reverse T3. We successfully analyzed the co-expression between Thra and Slc16a2 in 54 cell types and between Thrb and Slc16a2 in 40 cell types (**Table S2**). With p <0.05, we found Thra and Thrb both tended to be co-expressed with Slc16a2 in hepatocytes, endothelial cells, neurons and brain pericytes, which suggests they exhibit high capability of thyroid hormone transport through Slc16a2 for the Thra- and Thrb- expressing cells. We successfully analyzed the co-expression between Thra and Slc16a10 in 53 cell types and between Thrb and Slc16a10 in 42 cell types. With p <0.05, we found Thra and Thrb both tended to be co-expressed with Slc16a10 in hepatocytes, intestinal crypt stem cells, mesenchymal stem cells of adipose tissue and atrial myocytes, which suggests they exhibit high capability of thyroid hormone transport through Slc16a10 for the Thra- and Thrb- expressing cells. With single-cell RNA-Seq data, we could study the co-expression between TRs genes and transports and transporters (or deiodinases) in individual cells. Please find more detailed results of each cell type in **Table S2**.

Using the single-cell RNA-seq data, we are able to analyze TRs genes expression as well as the co-expression between the TR genes and thyroid hormones transporters or deiodinases in the cell types. This provides vital information in understanding molecular mechanisms of thyroid hormone regulation diseases and in the identification of potential cells in tissues and organs to target with drugs.

### Thra and Thrb Expression in Cell Subtypes From Three-Month-Old Mice

Taking 0.2 as a threshold, we found 78 of the above 101 cell types showed low Thra/Thrb expression. Employing single-cell RNA-Seq technology, a remarkable process in recognizing the subtypes of different immune cells and non-immune cells has been made (24, 25). This reminded us that the cells comprising a cell type may be highly heterogeneous and encouraged us to focus on these cell types to determine the subtypes with significant Thra/Thrb expression.

**Figure 2:**
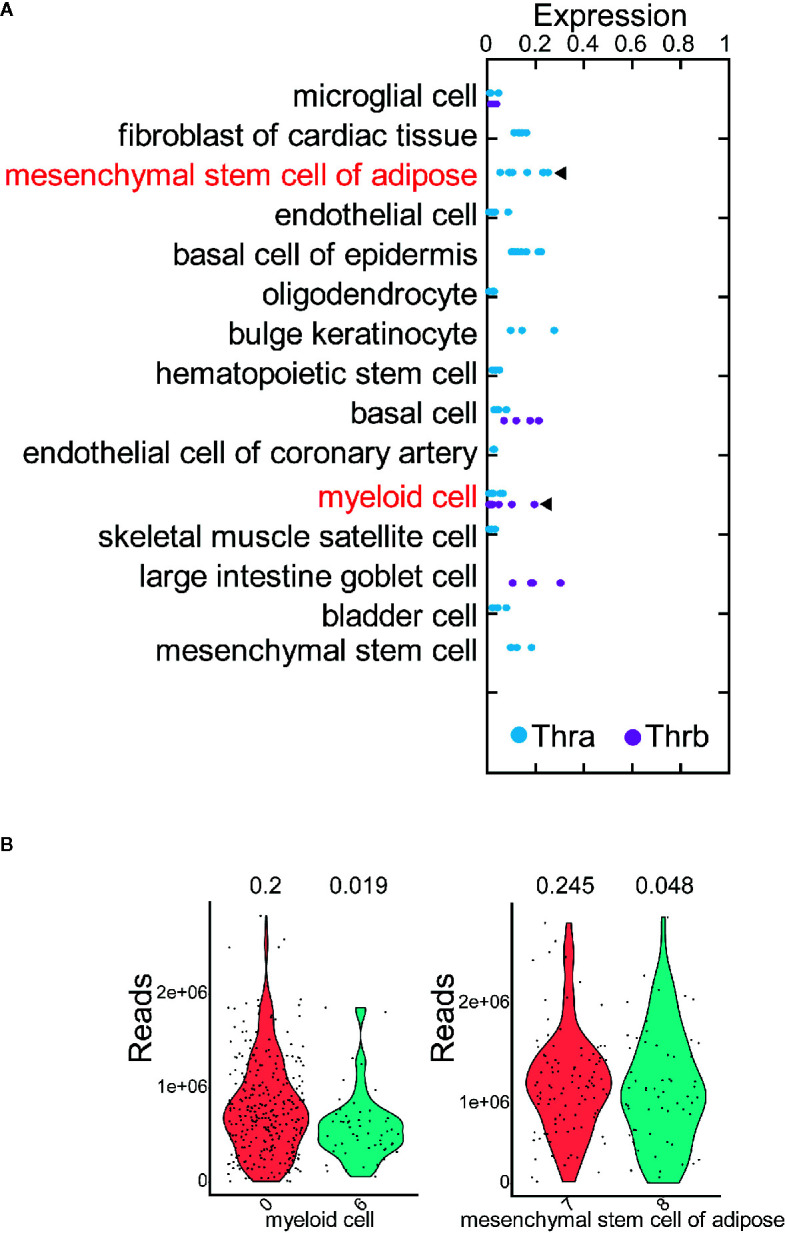
The expression of Thra and Thrb in different cell clusters. **(A)** The expression percentage of Thra or Thrb in the cell clusters from the 15 selected cell types. The cell types whose cell clusters showing the significant variance of Thra/Thrb expression percentages are highlighted. **(B)** Sequencing depth of cells from the selected cell clusters. The X-axis indicates the index of the cell cluster.

**Figure 3:**
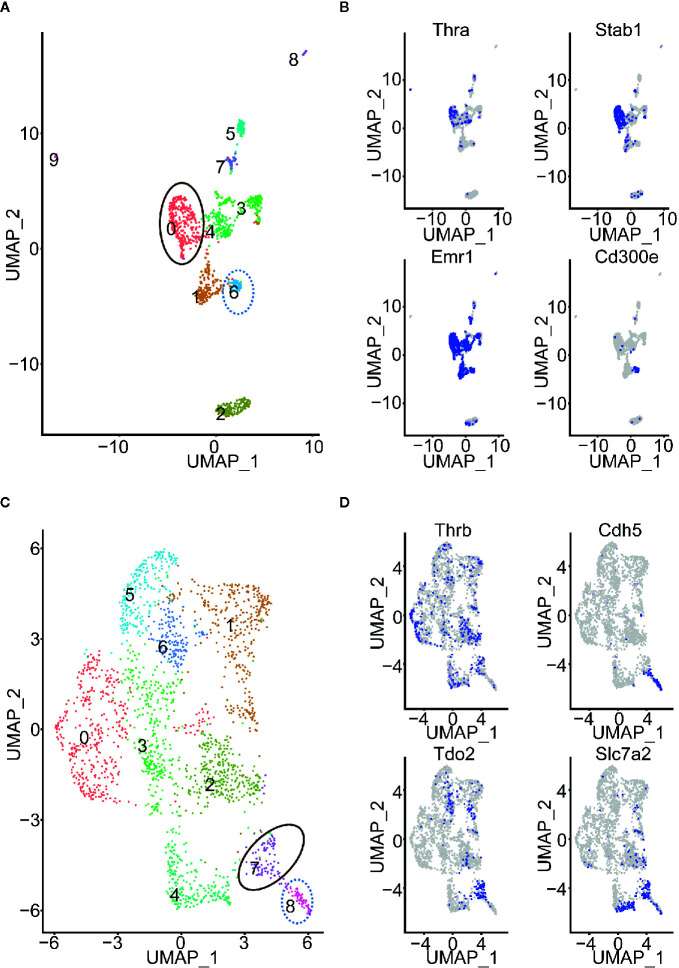
Cell clusters of myeloid cells and mesenchymal stem cells of adipose tissue. **(A)** Cell clusters of myeloid cells. The cell clusters with high or low Thra expression percentages are indicated. **(B)** The expression of Thra, Stab1, Emr1, and Cd300e in the cell clusters of the myeloid cells. **(C)** The cell clusters of mesenchymal stem cells of adipose tissue. The cell clusters with high or low Thrb expression percentages are indicated. **(D)** The expression of Thrb, Cdh5, Tdo2, and Slc7a2 in the cell clusters of the mesenchymal stem cells of adipose tissue are indicated.

We selected 15 of the 78 cell types, in which: at least 500 cells are profiled; more than 50 cells expressed Thra (or Thrb); and either Thra expression or Thrb expression is less than 0.2 (**Table S1**). Thus, we obtained a sufficient number of cells to conduct the cell clustering analysis and evaluate Thra/Thrb expression in the cell types. In each cell type, we grouped the cells into cell clusters according to their gene expression profiles using Seurat (26) (see *Using the Seurat Package to Group Cells into Cell Clusters and Identify Marker Genes for Each Cluster*” in the *Materials and Methods*). We then counted the cells expressing Thra/Thrb in each cell cluster and calculated the percentage of cells expressing the gene. We discovered that, in the myeloid cell, there was a large variance in the percentage of Thra expression among the cell clusters (**Figure 2A**). In the mesenchymal stem cells of adipose tissue, the cell clusters have different Thrb expression percentages. We calculated the change in Thra (or Thrb) expression percentage between the cell clusters and selected the two cell clusters with a more than a five-fold change in the two cell types. We calculated the sequencing depth of each cell, and compared the sequencing depth of cells from the two cell clusters (**Figure 2B**). We found that the sequencing depth of the two selected clusters is comparable in myeloid cells and mesenchymal stem cells of adipose tissue. We visualized the cell clusters in the myeloid cell with the uniform manifold approximation and projection (UMAP) technique (see *Using the Seurat Package to Group Cells into Cell Clusters and Identify Marker Genes for Each Cluster* in the *Materials and Methods*, **Figure 3A**). We identified the marker genes for each cell cluster (**Table S3**). A GO term enrichment analysis of the marker genes showed that innate immune response related processes were activated in the clusters studied; cluster 0 had a Thra expression percentage of 0.2, and cluster 6 had a Thra expression percentage of 0.019 (**Table S4**). We also found that both cluster 0 and cluster 6 expressed Emr1 at a high level, which is a marker for monocyte/macrophage cells (27) (**Figure 3B**). However, cluster 0 specifically expressed Stab1, and cluster 6 uniquely expressed Cd300e. The gene annotation from the Refseq database (28) showed that Cd300e encodes the CD300 glycoprotein, which is a member of the cell surface protein family expressed on myeloid cells in humans. The protein interacts with the TYRO protein tyrosine kinase binding protein and is thought to act as an activating receptor. Stab1 encodes a large, transmembrane receptor protein that may function in angiogenesis, lymphocyte homing, cell adhesion, or receptor scavenging in humans. Previous work reported that Stab1 expression defines a subset of macrophages that mediate tissue homeostasis and prevent fibrosis in chronic liver injury (29). The two cell clusters may belong to different monocyte/macrophage subtypes, and the Stab1 marker subtype may be more sensitive to thyroid hormone regulation through Thra.

We visualized the cell clusters in mesenchymal stem cells of adipose tissue using the UMAP technique (**Figure 3C**). The marker genes of each cell cluster were identified (**Table S5**). The GO term enrichment analysis of the marker genes showed the “response to hypoxia” process is activated in the clusters studied: cluster 7 had a Thrb expression percentage of 0.245, and cluster 8 had a Thrb expression percentage of 0.048 (**Table S6**). Previous work has demonstrated that human adipose-derived mesenchymal stem cells (hASCs) were found to reside in a relatively low oxygen tension microenvironment in the body (30). Cluster 7 specifically expressed both Tdo2 and Slc7a2, while cluster 8 uniquely expressed Cdh5 (**Figure 3D**). The Tdo2 and Slc7a2 marker subtype were more sensitive to thyroid hormone regulation through Thrb.

The cell subtype analysis described here is intuitive and requires further validation. However, it demonstrates that cells within a cell type may be heterogeneous and respond to thyroid hormone regulation with various sensitivities. To capture the potential sensitive subtypes, it is worth focusing on the cell types that have low Thra or Thrb expression but are heterogeneous.

### Integration of Gene Expression Profiles at the Cell Type and Tissue Levels to Identify the Mouse Liver Cells Responsive to T3 Treatment

Multiple methods of chemical stimulation and genetic manipulation have been conducted on mice to understand the mechanism of thyroid hormone regulation under different conditions. Direct single-cell RNA sequencing aids in the accurate discovery of responsive cells. However, currently, the cost of single-cell RNA sequencing is approximately 30 times higher than bulk tissue RNA sequencing. It is unaffordable for wet lab researchers who design their experiments to include multiple treatment groups with multiple mice enrolled in each group. Here, we demonstrated an approach to integrate the single-cell gene expression profiles generated by the Tabula Muris Consortium and the bulk tissue gene expression profiles generated by wet lab researchers to identify cells that are potentially responsive to thyroid hormone treatment in tissues.

Finan et al. developed a euthyroid mouse liver model using a 16-day T3 treatment on six to 12 month-old C57Bl/6j mice (31). Here, as this work recommends, we detected the up-regulated and down-regulated genes following T3 treatment using DESeq2 (32) (see *Gene Differential Expression Analysis* in the *Materials and Methods*, **Table S7**). Yuan et al. developed mouse liver models (euthyroid or hypothyroid), using a one-day T3 treatment on nine-weeks-old C57Bl/6j mice (33). Their work listed the induced (up-regulated) and repressed (down-regulated) genes following T3 treatment on the euthyroid and hypothyroid livers (**Table S8**).

**Figure 4:**
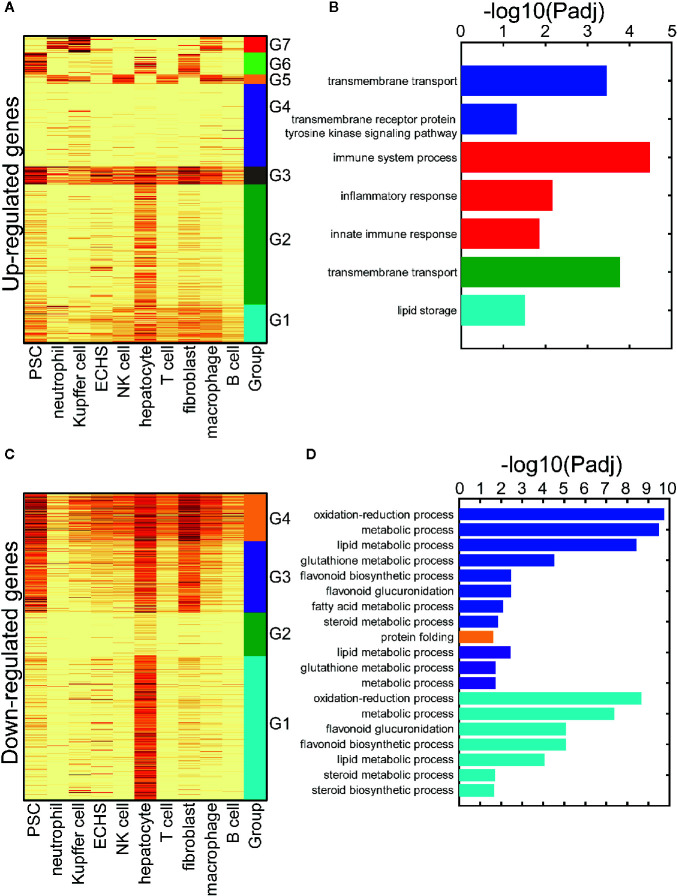
The cell-type-specific patterns of the up-and down-regulated genes following a long-term T3 treatment. **(A)** The cell-type-specific patterns of the up-regulated genes. PSC: pancreatic stellate cell; ECHS: endothelial cell of the hepatic sinusoid. **(B)** The significant GO terms of the up-regulated genes. The GO terms of all the up-regulated genes are listed and marked by the blue bars. The parts of the up-regulated genes, which are specifically expressed in some cell-type groups, are listed and marked with the same color as the groups in panel **(A, C)** The cell-type-specific patterns of the down-regulated genes. PSC, pancreatic stellate cell; ECHS, endothelial cell of the hepatic sinusoid. **(D)** The significant GO terms of the down-regulated genes. The GO terms of all the down-regulated genes are listed and marked by the blue bars. The parts of the down-regulated genes, which are specifically expressed in some cell-type groups, are listed and marked with the same color as the groups in panel **(C)**.

Again, we simulated the cell microenvironment of the liver using ten cell types from the SMART-Seq2 platform in three-month-old mice, which include: natural killer (NK) cells, B cells, T cells, neutrophils, macrophages, Kupffer cells, hepatocytes, endothelial cells of the hepatic sinusoid, fibroblasts, and, pancreatic stellate cell. We conducted a hierarchical clustering analysis on the up-regulated genes from work reported by Finan et al. using their expression profiles in the ten cell types (see *Clustering Analysis of the DEGs* in the *Materials and Methods*). The up-regulated genes are decomposed into seven gene clusters (clusters G1–G7 in **Figure 4A**). Cluster G1 includes genes exhibiting moderate expression in the ten cell types. Cluster G2 contains genes with specific, high expression in hepatocytes. The genes expressed at a high level in the pancreatic stellate cells, endothelial cells of the hepatic sinusoid, hepatocytes, and fibroblasts are in the cluster G3. Cluster G4 incorporates genes with low expression in the ten cell types. The genes in cluster G5 are specifically expressed in immune cells, while the genes specifically expressed in pancreatic stellate cells, hepatocytes, and fibroblasts are in cluster G6. The genes in cluster G7 exhibit specific expression in neutrophils, Kupffer cells, and macrophages. Breakdown of the decomposed up-regulated genes improved our understanding of the up-regulated gene regulation in cell types that respond to T3. We also detected the enriched GO terms in the up-regulated gene set and in the decomposed G1–G7 gene clusters (**Figure 4B**). The up-regulated genes indicated activation of the transmembrane cell transport function in the liver tissue, which was also revealed by the genes in cluster G2. However, the up-regulated genes did not indicate that the innate immune response was evoked in the myeloid-derived cells (neutrophils, Kupffer cells, and macrophages) following long-term T3 treatment.

We also conducted a hierarchical clustering analysis on the down-regulated genes in a similar way. The genes were decomposed into four gene clusters (clusters G1–G4 in **Figure 4C**). Cluster G1 contains genes with specific, high expression in hepatocytes. The genes in cluster G2 exhibited weak expression in the ten cell types. The genes expressed at a high level in pancreatic stellate cells, hepatocytes, and fibroblasts were in cluster G3. Cluster G4 included the genes widely expressed in all ten cell types. As above, we detected the enriched GO terms in the down-regulated gene set and the decomposed G1–G4 gene clusters (see *GO Term Enrichment Analysis* in the *Materials and Methods*, **Figure 4D**). The down-regulated differentially expressed genes (DEGs) indicated disruption of the metabolic process in the liver cells. However, the GO term results of the cluster G1 genes clarifies that this occurs in the hepatocytes.

**Figure 5:**
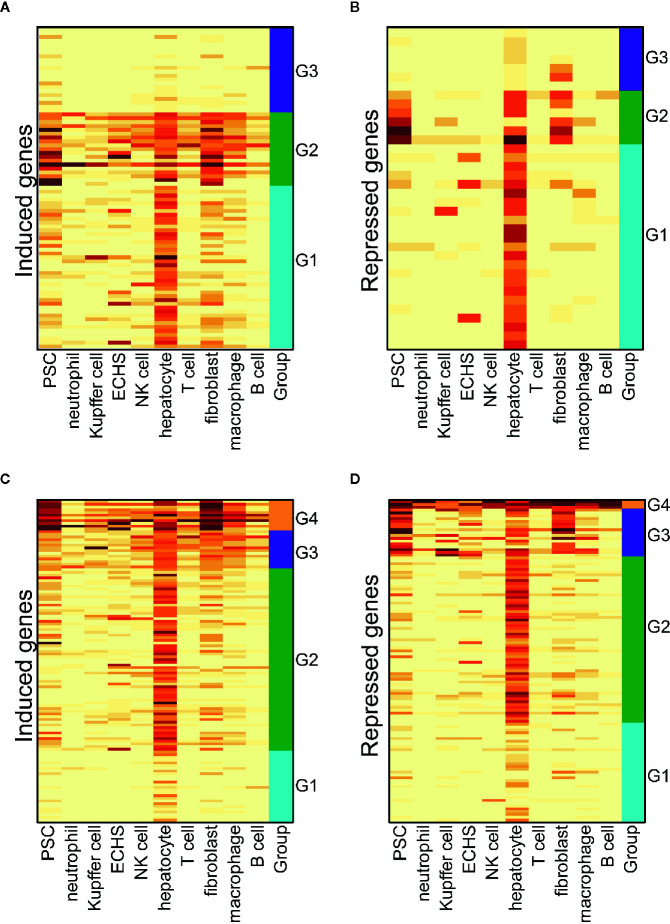
The cell-type-specific patterns of the induced and repressed genes flowing a short-term T3 treatment. **(A)** The cell-type-specific patterns of induced genes in the euthyroid liver. PSC, pancreatic stellate cell; ECHS, endothelial cell of the hepatic sinusoid. **(B)** The cell-type-specific patterns of repressed genes in the euthyroid liver. **(C)** The cell-type-specific patterns of induced genes in hypothyroid liver. **(D)** The cell-type-specific patterns of repressed genes in the hypothyroid liver.

We conducted a hierarchical clustering analysis on the induced and repressed genes following short-term T3 treatment. It is clear that, in either the euthyroid liver or hypothyroid liver, the most dysregulated genes are expressed at high levels in hepatocytes, suggesting that hepatocytes are most responsive to T3 treatment (**Figures 5A–D**). We also detected the enriched GO terms in the induced or repressed genes and gene clusters shown in **Figure 5** (see *GO Term Enrichment Analysis*” in the *Materials and Methods*). We found no significant GO terms in the induced and repressed genes in either the euthyroid or hypothyroid mouse liver. However, the oxidation-reduction process was enriched in the G2 gene cluster of the induced genes following T3 treatment in the hypothyroid mouse liver (**Figure 5C**).

The results reveal that long-term T3 treatment may have a broad impact on multiple cell types, and hepatocytes are the primarily responsive cells. Short-term T3 treatment may have a limited impact, especially on hepatocytes. The oxidation-reduction process is activated in the hepatocytes of a hypothyroid mouse following short-term T3 treatment, which suggests a normal thyroid hormone regulation function (34). However, coupled with various other metabolic processes, this process is inhibited in hepatocytes of a euthyroid mouse following long-term T3 treatment (**Figures 4C, D**), which implies excess thyroid hormone regulation may impair hepatocyte function. The evoked innate immune response in the myeloid-derived cells (neutrophils, Kupffer cells, and macrophages) may damage hepatocytes. Our previous analysis (*Thra and Thrb Expression in 101 Cell Types From Three-Month-Old Mice*) showed an extremely low expression of TRs genes in the immune cells of the liver, and that hepatocytes have an obvious, higher level of Thrb expression compared to that of Thra (**Figure 1**). It is suggested that hepatocytes may respond to T3 treatment *via* Thrb and that the immune cell response is largely evoked by paracrine signaling in hepatocytes.

The above results are derived from *in silico* analyses and further validation using wet lab experiments is required. However, the results demonstrate that gene expression profiling at bulk tissue RNA-Seq level followed by decomposition of dysregulated genes into different cell types assists in the understanding of the impact of thyroid hormones on different cell types.

## Discussion

Thyroid hormones mediate a remarkable range of functions all over the human body. Previous tissue-specific analyses of Thra and Thrb gene expression identified the potential biological response in tissues and organs. However, tissues and organs are made up of billions of multiple cell types. The mechanism by which cell types respond to thyroid hormone regulation, and which are the primary responsive cells is a critical question in understanding the molecular mechanism of thyroid hormone regulation diseases.

Single-cell RNA sequencing technology is a powerful tool used to profile gene expression programs in individual cells. It was developed in 2009 and became commercially available in the last five years. It provides an unprecedented opportunity to recognize the cell types comprising the tissues and organs and investigate their response to thyroid hormone regulation. Here, we employed the compendium of mouse single-cell transcriptome data to trace the responsive cell types and subtypes in tissues. Ranking the 101 cell types by the level of Thra expression indicated the top five cell types are oligodendrocytes, medium spiny neurons, neuronal stem cells, neurons, and brain pericytes, which may explain why Thra has a significantly high expression in the brain. We analyzed the cell types comprising the liver and found fibroblasts had the highest levels of Thra expression while hepatocytes had the highest Thrb expression, which suggests they are potentially the most sensitive cell type for thyroid hormone regulation through Thra and Thrb. In addition, we analyzed the expression profiles of transporters and deiodinases and found co-expression of transporters and deiodinases with TRs genes in different cell types. For cell types with low TRs gene expression, we focused on the cell types comprised of heterogeneous cells to find the potential subtypes sensitive to thyroid hormone regulation. Thra expression is low in myeloid cells. However, we found a Stabl marker subtype with moderate Thra expression, which suggested it is the most sensitive for thyroid hormone regulation through Thra. Employing the gene expression profiles of each cell type, we decomposed the DEGs in the mouse liver tissue RNA-Seq data following either short-term or long-term T3 treatment into different cell types and identified the responsive cell types. Hepatocytes are the primary responsive cells, and possibly respond to the treatment through Thrb. All the above results are derived from *in silico* analyses, and need to be further validated using wet lab experiments. However, we believe the results will attract the interest of wet lab researchers and encourage collaboration between *in silico* researchers and wet lab researchers, allowing full advantage to be taken of the compendium of single-cell RNA-Seq data released by Tabula Muris Consortium.

In addition to the organs belonging to the endocrine system, many other organs that are part of other systems in the human body have secondary endocrine functions, including the bone, kidneys, liver, heart, and gonads. For example, hepatocytes in the liver secrete hepcidin, which is a key regulator for the entry of iron into the circulation in mammals (35). These hormones are delivered to distant organs through the circulatory system and regulate the cells targeted. In this work, we demonstrated approaches to trace the cells responsive to hormones using the single-cell RNA-Seq data from tissues and organs of the entire human body. Recently, Han et al. used single-cell RNA sequencing to determine the cell-type composition of all major human organs and constructed a scheme for the human cell landscape (13). We used the gene expression profiles of mouse cells from the Tabula Muris Consortium; the gene expression profiles of the human cells in Han’s work were not used because of the impressive sequencing depth provided by the Tabula Muris Consortium. The more genes we can detect, the more cell type features we can capture. We believe our proof-of-concept work reported here paves the way to the largescale screening of the function of hormones in human cell types, which will lead to the dramatic improvement in our understanding of hormone mechanisms.

## Data Availability Statement

Publicly available datasets were analyzed in this study. All the data are publicly available from the GEO database (https://www.ncbi.nlm.nih.gov/geo/). The single-cell RNA-Seq data and coupling cell annotation file can be found under the accession code GSE149590; the bulk RNA-Seq data for the 17 tissues and organs and associated sample information file can be found under the accession code GSE132040; the liver tissue RNA-Seq data for long-term T3 treated samples and the controls can be found under the accession code GSE85793.

## Author Contributions

LH and CW contributed to the conception and design of the study. CW organized the database. CW performed the statistical analysis. LH and CW drafted the manuscript. All authors contributed to the article and approved the submitted version.

## Funding

CW is supported by the National Program on Key Research Project of China (2017YFA0104900) and the Independent Task of State Key Laboratory for Diagnosis and Treatment of Infectious Diseases.

## Conflict of Interest

The authors declare that the research was conducted in the absence of any commercial or financial relationships that could be construed as a potential conflict of interest.

## Supplementary Material

Supplementary Figure 1 Thra and Thrb expression in the cells sequenced by SMART-Seq2 platform. **(A)** Thra and Thrb expression in the 78 cell types from three-,18-, and 24-month-old mice. **(B)** Pearson’s correlation coefficients of Thra (or Thrb) expression between different ages of mice. 3, 18, and 24 m represent the 3-, 18-, and 24-month-old mice. All coefficients satisfy p-value <0.05.

Supplementary Figure 2 Thra and Thrb expression in the cells sequenced by 10x Genomics platform. **(A)** Thra and Thrb expression in the 30 cell types from 1-, 3-, 18-, 21-, 24-, and 30-month-old mice. **(B)** Pearson’s correlation coefficients of Thra (or Thrb) expression between different ages of mice. 1, 3, 18, 21 24, and 30 m represent the 1-, 3-, 18-, 21-, 24-, and 30-month-old mice. All coefficients satisfy p-value <0.05.

Supplementary Figure 3 Thra and Thrb expression in the cell types sequenced by both SMART-Seq2 and 10× Genomics platforms in three-, 18- and 24-month-old mice. 3, 18, and 24m represent three-, 18-, and 24-month-old mice. Pearson’s correlation coefficients (rho) of Thra (or Thrb) expression between mice sequenced by SMART-Seq2 and 10× Genomics platforms were calculated. All coefficients satisfy p-value <0.05.

Supplementary Figure 4 Thra and Thrb expression in 29 cell types uniquely sequenced by 10× Genomics platform in 3-month mice.

Supplementary Figure 5 The expression of Slc16a2, Slc16a10, Slco1c1 and Dio1, Dio2, Dio3 in 101 cell types from 3-month-old mice.
