# Supplementary figures and images for: Using Single-Cell RNA-Seq Data to Trace Tissue Cells Responsive to Thyroid Hormones
Source: Front Endocrinol (Lausanne). 2021 Feb 24;12:609308. doi: 10.3389/fendo.2021.609308 (PMC7943891; doi:10.3389/fendo.2021.609308)

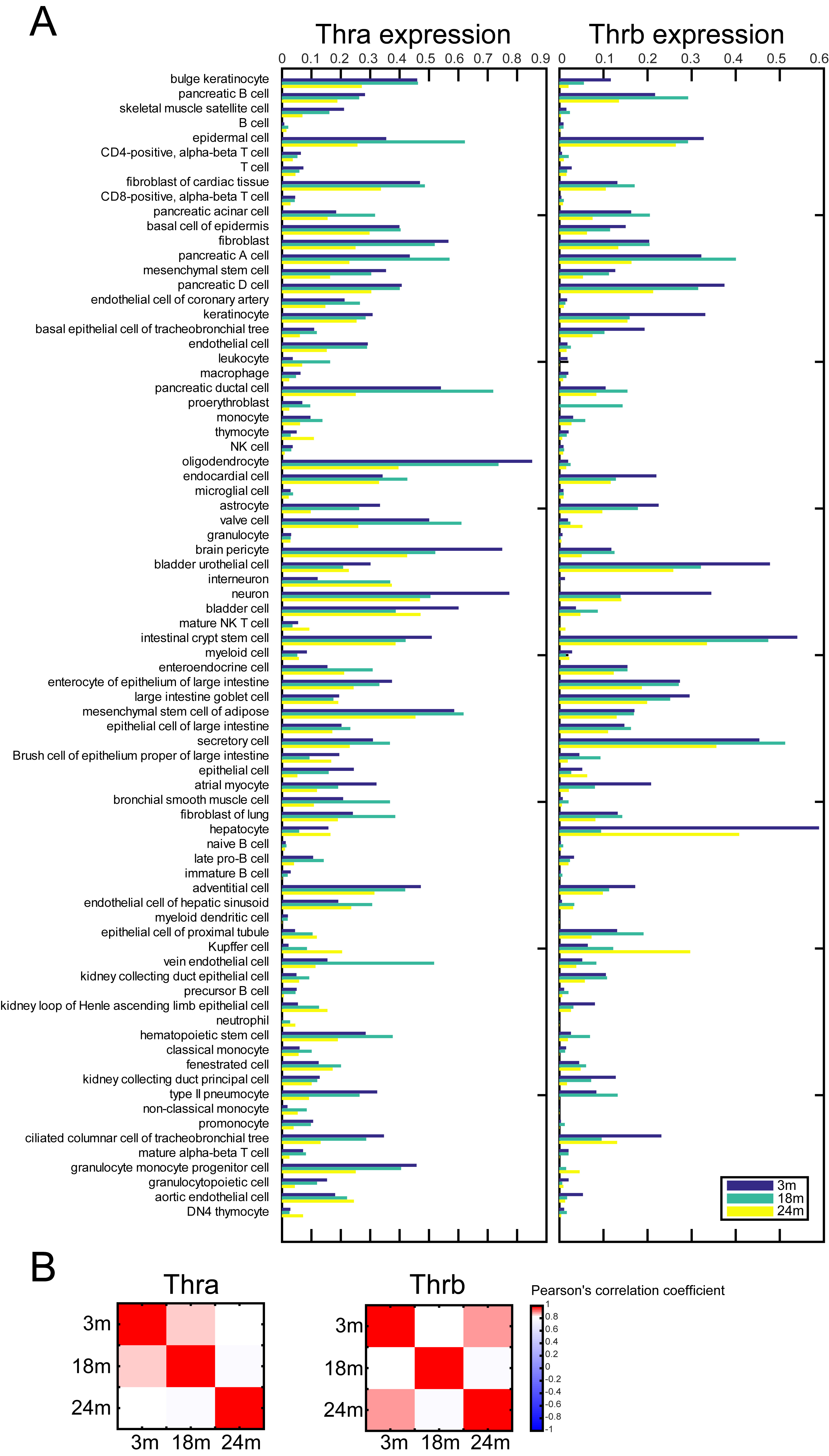

Supplement: Supplementary Figure 1 — Thra and Thrb expression in the cells sequenced by SMART-Seq2 platform. (A) Thra and Thrb expression in the 78 cell types from three-,18-, and 24-month-old mice. (B) Pearson’s correlation coefficients of Thra (or Thrb) expression between different ages of mice. 3, 18, and 24 m represent the 3-, 18-, and 24-month-old mice. All coefficients satisfy p-value <0.05. [file Image_1.jpeg]

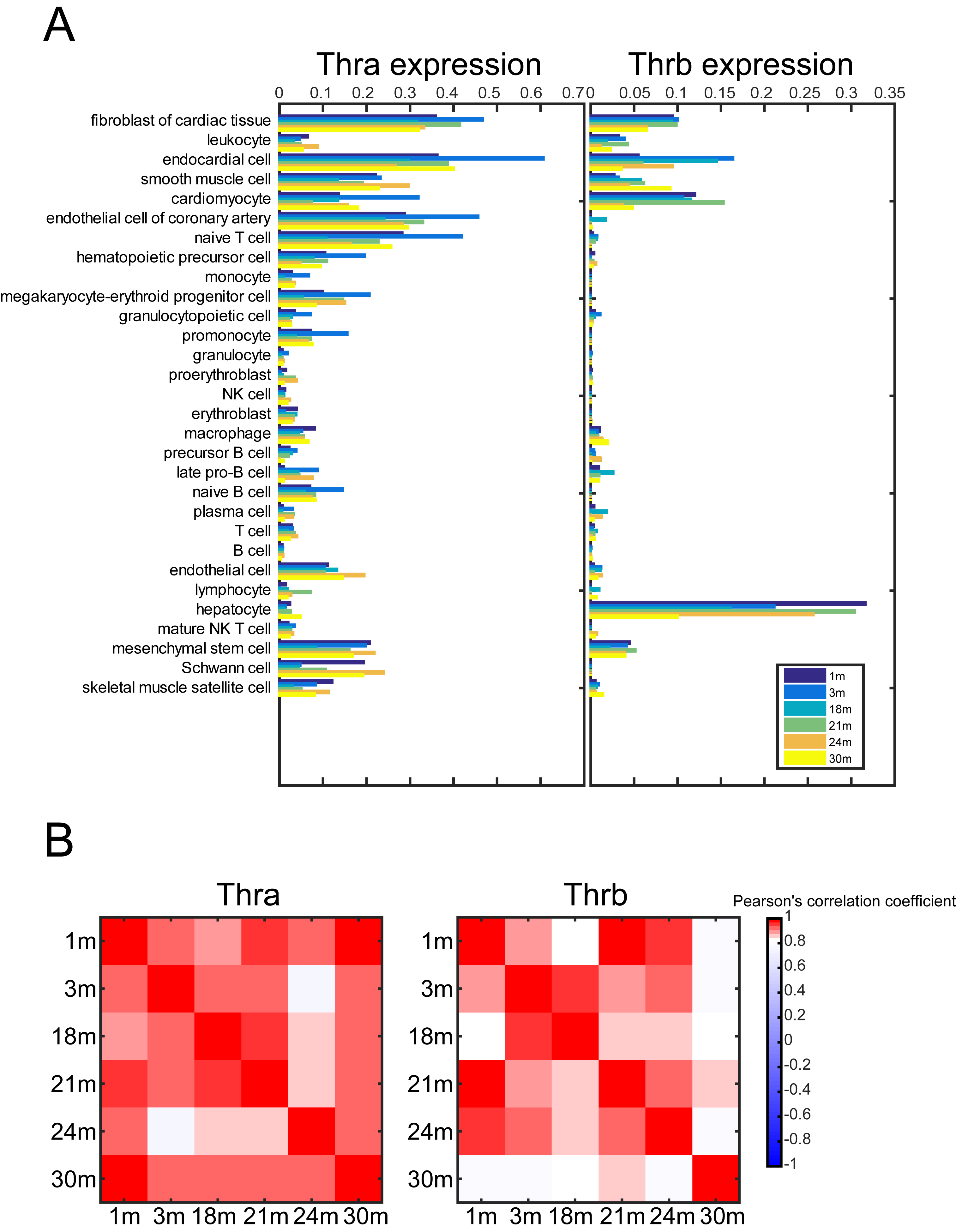

Supplement: Supplementary Figure 2 — Thra and Thrb expression in the cells sequenced by 10x Genomics platform. (A) Thra and Thrb expression in the 30 cell types from 1-, 3-, 18-, 21-, 24-, and 30-month-old mice. (B) Pearson’s correlation coefficients of Thra (or Thrb) expression between different ages of mice. 1, 3, 18, 21 24, and 30 m represent the 1-, 3-, 18-, 21-, 24-, and 30-month-old mice. All coefficients satisfy p-value <0.05. [file Image_2.jpeg]

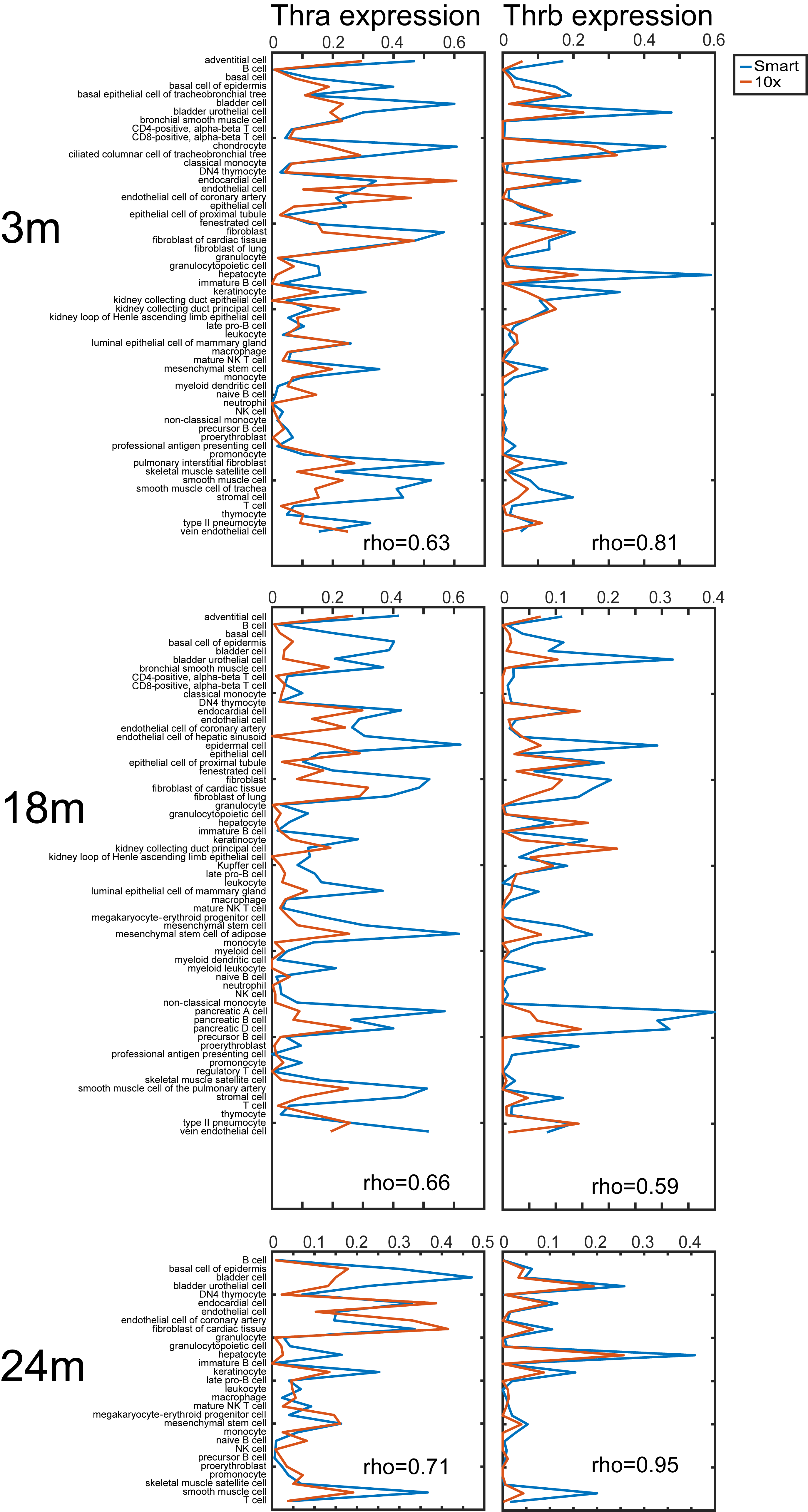

Supplement: Supplementary Figure 3 — Thra and Thrb expression in the cell types sequenced by both SMART-Seq2 and 10× Genomics platforms in three-, 18- and 24-month-old mice. 3, 18, and 24m represent three-, 18-, and 24-month-old mice. Pearson’s correlation coefficients (rho) of Thra (or Thrb) expression between mice sequenced by SMART-Seq2 and 10× Genomics platforms were calculated. All coefficients satisfy p-value <0.05. [file Image_3.png]

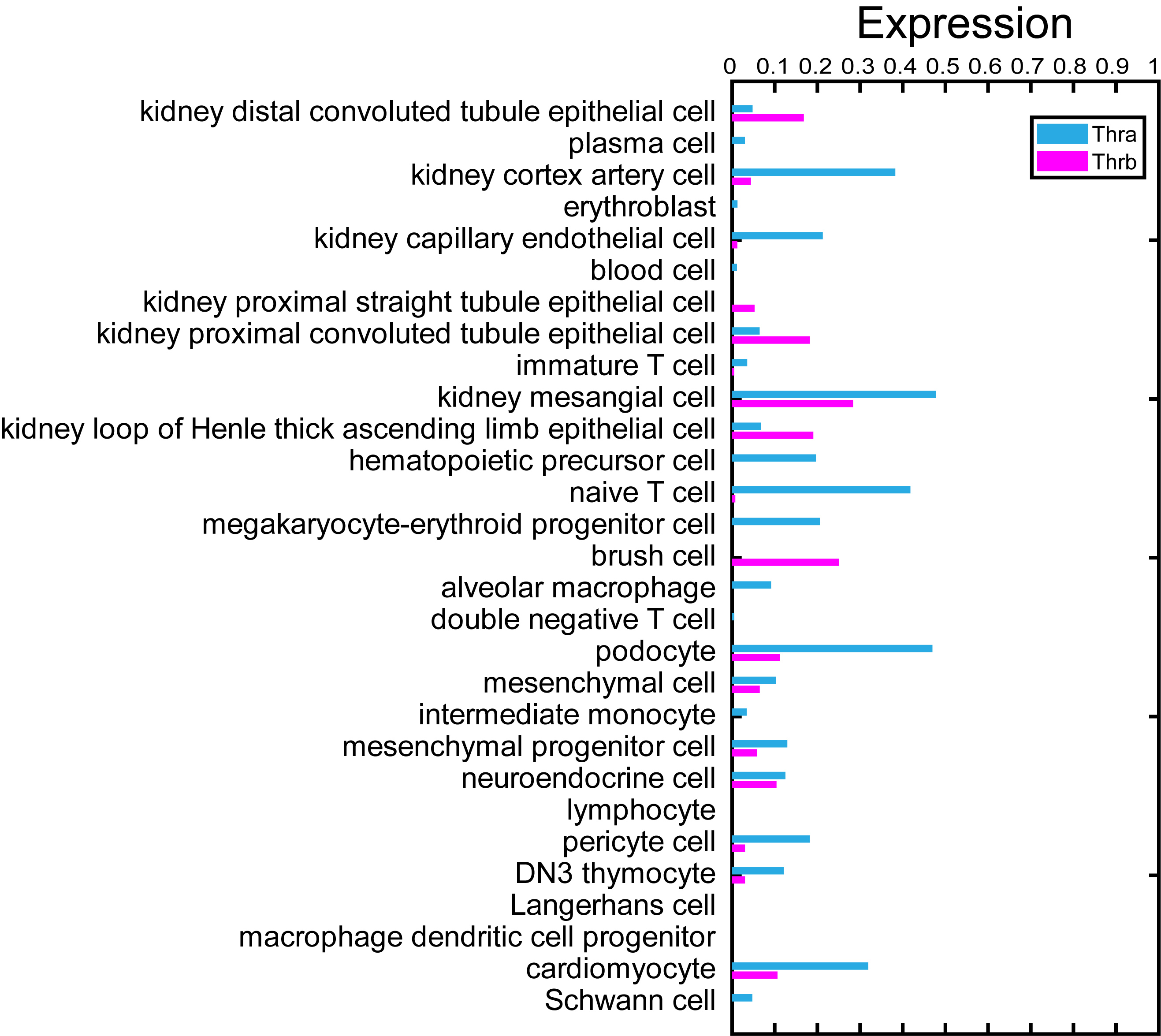

Supplement: Supplementary Figure 4 — Thra and Thrb expression in 29 cell types uniquely sequenced by 10× Genomics platform in 3-month mice. [file Image_4.jpeg]

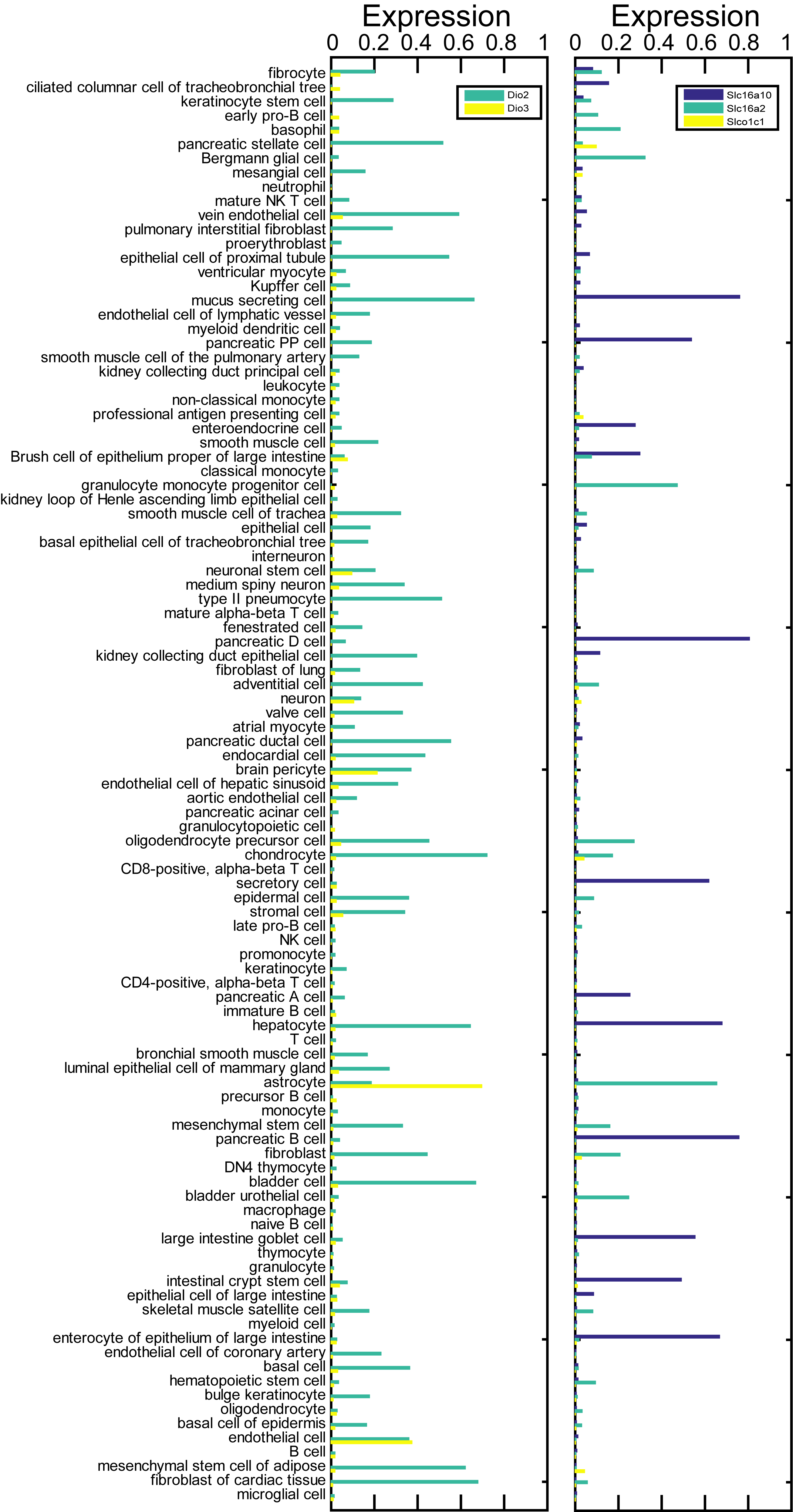

Supplement: Supplementary Figure 5 — The expression of Slc16a2, Slc16a10, Slco1c1 and Dio1, Dio2, Dio3 in 101 cell types from 3-month-old mice. [file Image_5.png]
